# The chromatin-remodeling enzyme BRG1 promotes colon cancer progression via positive regulation of WNT3A

**DOI:** 10.18632/oncotarget.13326

**Published:** 2016-11-12

**Authors:** Shengtao Lin, Tao Jiang, Ling Ye, Zhongbo Han, Yuan Liu, Chenchen Liu, Chenwei Yuan, Senlin Zhao, Jian Chen, Jingtao Wang, Huamei Tang, Su Lu, Liguang Yang, Xiaoliang Wang, Dongwang Yan, Zhihai Peng, Junwei Fan

**Affiliations:** ^1^ Department of General Surgery, Shanghai General Hospital, Shanghai Jiao Tong University School of Medicine, Shanghai, 200080,China; ^2^ Department of Anal-Colorectal Surgery, General Hospital of Ningxia Medical University, Yinchuan 750004, China; ^3^ Department of General Surgery, Central Hospital of Zi Bo, Zi Bo, Shandong 255000, China; ^4^ Department of Pathology, Shanghai General Hospital, Shanghai Jiao Tong University School of Medicine, Shanghai 200080, China; ^5^ Key Lab of Systems Biology, Shanghai Institutes for Biological Sciences, Chinese Academy of Sciences, Shanghai 200031, China

**Keywords:** BRG1, colon cancer, progression, WNT3A

## Abstract

In this study, we aimed to elucidate the clinical significance and underlying mechanisms of BRG1 in colon cancer. In the clinical analysis, overexpression of BRG1 correlates with colon cancer progression in two cohorts (*n* = 191 and *n* = 75). Kaplan-Meier survival analysis revealed that BRG1 is a prognosis predictor for overall survival (*P* < 0.001) and disease-free survival (*P* = 0.001). Knocking down BRG1 expression significantly suppressed the proliferation and invasion in colon cancer cells. The expression pattern of WNT3A is consistent with BRG1 in colon cancer tissues and WNT3A expression was inhibited in BRG1 knockdown cells. In addition, restoring WNT3A expression rescues the inhibition of cell proliferation and invasion induced by BRG1. In this study, we demonstrate that BRG1 may contribute to colon cancer progression through upregulating WNT3A expression.

## INTRODUCTION

Colorectal cancer is the third most common type of cancer worldwide and the fourth most common cause of death [1]. In China and other developing countries, the incidence of colon cancer has increased in recent years and it has become a substantial cancer burden. The pathogenesis of colon cancer is heterogeneous and the occurrence of colon cancer contains a series of molecular events that result from an accumulation of genetic and epigenetic changes in colon epithelial cells [2]. Previous studies have validated that mutations in certain genes (*APC, KRAS, TP53 BRAF*), chromosomal instability, and other genetic changes play vital roles in the multistep process of carcinogenesis in colon cancer [2, 3]. In recent years, DNA methylation, histone modification, and chromatin remodeling, together with other changes in epigenetics, were also proven to accelerate the formation and progression of colon cancer through regulation of gene expression [4, 5].

In eukaryotic cells, DNA is packaged into a highly compact chromatin structure and the fundamental packing unit is known as the nucleosome. Biochemical and genetic evidence indicates that nucleosomes normally represses transcription by physically preventing binding factors from accessing to the binding sites [6]. Chromatin remodelers dynamically alter the structures of chromatin to regulate target gene expression [7, 8]. The mammalian SWI/SNF complexes constitute a family of chromatin remodeling proteins that regulate gene expression by disrupting histone-DNA contacts in an ATP-dependent manner. The complexes are evolutionarily conserved in eukaryotes and contain either Brahma (BRM) or Brahma-related gene 1 (BRG1) as their central ATPase subunit [9]. The increased expression of BRG1 acts as a suppressor in some kinds of tumors, including pancreatic adenocarcinoma, skin cancer, and lung cancer [10–12]. However, Liu et al. reported that BRG1 promotes chemoresistance of pancreatic cancer cells [13]. The controversial effects of BRG1 in pancreatic cancer may imply different function of BRG1 in certain circumstance. In addition, the increased expression of BRG1 is associated with the development and progression of breast cancer, gastric cancer, and melanoma [14–16]. These findings suggest that the biological significance of SWI/SNF chromatin remodeling complexes differs during the pathogenesis of human cancer according to the cell and/or tissue type [17, 18]. So, it’s of great significance to elucidate the role of BRG1 in colon cancer.

As a transcription regulation factor, BRG1 participates in colon cancer progression through regulating its target gene. We choose WNT3A as the target gene of BRG1 in this study for three reasons: First, WNT3A was identified as potential downstream gene of BRG1 in colon cancer cell using Agilent Whole Human Genome Oligo Microarray, iReport (www.qiagen.com/ingenuity, Ingenuity Systems, Inc.) and QIAGEN’s Ingenuity Pathway Analysis software; Second, WNT3A promoted the vasculogenic mimicry formation of colon cancer via Wnt/β-catenin signaling and was associated with epithelial-mesenchymal transition (EMT), so WNT3A contributed to colon cancer progression [19, 20]; Third, BRG1 had been proposed to be associated with β-catenin and Wnt/β-catenin signaling in previous research [21] and WNT3A is the representative canonical Wnt ligand [22].

In this study, we performed immunostaining of BRG1 on commercial colon cancer tissue microarray (TMA, *n* = 75) and our custom TMA (*n* = 191), and then explored the relationship between BRG1 protein expression and clinicopathological features of colon cancer. In addition, we knocked down BRG1 expression by RNA interference to probe the effects of BRG1 on biological behavior in 2 colon cell lines. The CCK-8 cell proliferation assay, clone formation assay, transwell invasion assay, and xenograft model were performed to analyze the influence of BRG1 on proliferation, and invasion. Moreover, the regulation effect of BRG1 on WNT3A expression was investigated in clinical samples and *in vitro*.

## RESULTS

### Overexpression of BRG1 in colon cancer tissues

**Figure 1:**
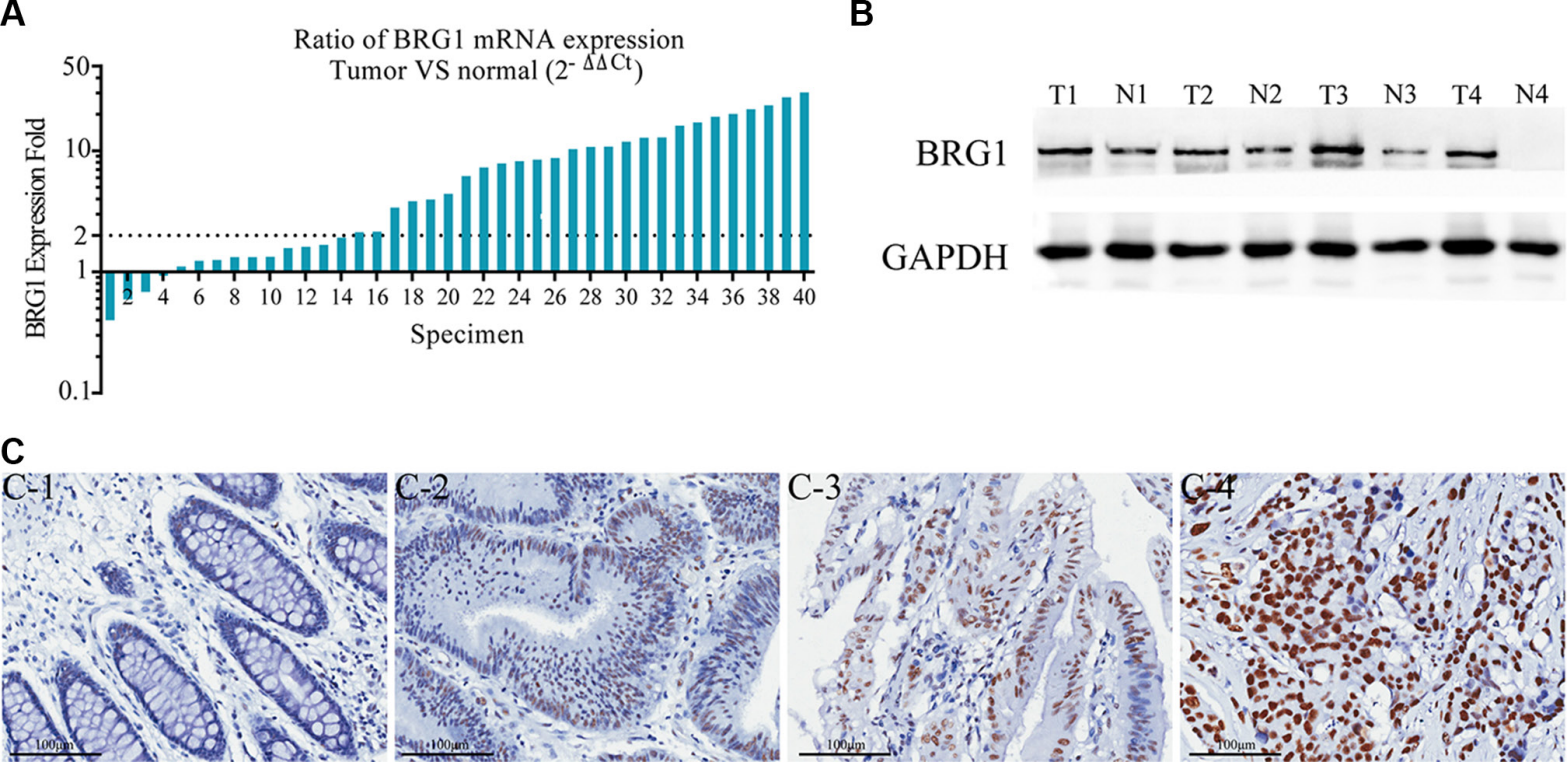
BRG1 overexpression in colon cancer tissues (**A**) Real-time PCR analysis of BRG1 mRNA in 40 paired colon cancer tissues. The logarithmic scale 2^-ΔΔCt^ was used to measure the relative BRG1 expression. BRG1 expression is increased in 36 (90%) colon cancer tissues than paired noncancerous tissues. More than 2-fold increase of BRG1 mRNA compared to noncancerous tissue was found in 26 (65%) colon cancer tissues. (**B**) Representative Western Blot results of BRG1 protein in matched colon cancer and uninvolved colon specimens. “T” and “N” represented tumor tissues and normal adjacent tissues in the same patients respectively. (**C**) Representative immunohistochemical staining results of BRG1 in tissues, C-1: Negative BRG1 expression in normal colonic mucosa epithelium, C-2, C-3: Weak BRG1 expression in well differentiated colon cancer tissues, C-4: Strong BRG1 expression in moderately differentiated colon cancer tissue (200×, Bar = 100 μm).

In order to assess the expression of BRG1 in colon cancer, we performed qRT-PCR and Western-blot in 40 colon cancer tissues paired with adjacent normal colon mucosa. Of the 40 paired cases, 36 (90%) colon cancer tissues showed an increased expression of BRG1 than paired normal tissues. In addition, 26 (65%) colon cancer tissues showed an increase over two-fold at mRNA level (Figure 1A). Similarly, BRG1 was markedly overexpressed at the protein level in cancer tissues compared to matched normal mucosa (Figure 1B). We analyzed BRG1 expression in 191 paired colon cancer tissues and adjacent normal mucosae. Results revealed that 16.2% (31/191) normal mucosae while 38.7% (74/191) colon cancer tissues showed moderate and strong BRG1 expression (*P* < 0.01). As shown in Figure 1C, BRG1 staining was observed mainly in the nuclei of the cancer cells. Significantly stronger BRG1 staining was observed in the carcinoma compared to adjacent normal colon tissue.

### Clinicopathological significance of BRG1 expression in colon cancer

In this study, we detected the association between BRG1 expression and clinicopathological parameters in two independent groups of patients. As displayed in Table 1, 74 colon cancer tissues showed moderate or strong staining. No significant correlation were obtained between BRG1 expression and age, gender, or location. However, expression of BRG1 was highly correlated with T category (*P* = 0.004), N category (*P* = 0.001), M category (*P* < 0.001), AJCC stage (*P* < 0.001), differentiation (*P* = 0.017) and vessel invasion (*P* = 0.042). Similar results were obtained when we analyzed the clinicopathological significance of BRG1 in 75 cases. Expression of BRG1 was significantly associated with T classification (*P* =0.047) and AJCC stage (*P* = 0.020) (Supplementary Table 1). These results intensely indicated a relationship between BRG1 and colon cancer progression.

**Table 1:** Associations of BRG1 expression with clinicopathological features in colon cancer (*n* = 191)

Variables	BRG1 expression		*P* value*
	Negative and weak (*n* = 117)	Moderate and Strong (*n* = 74)	
Age, n (%)^1^			0.256
< 65 years	41 (35.0)	32 (43.2)	
≥ 65 years	76 (65.0)	42 (56.8)	
Gender, *n* (%)^1^			0.947
Male	48 (41.0)	30 (40.5)	
Female	69 (59.0)	44 (59.5)	
Location, *n*(%)^1^			0.403
Ascending	45 (38.5)	34 (45.9)	
Transverse	14 (12.0)	4 (5.4)	
Descending	12 (10.3)	9 (12.2)	
Sigmoid	46 (39.3)	27 (36.5)	
T category, *n*(%)^1^			0.004*
T1 + T2	24 (20.5)	4 (5.4)	
T3 + T4	93 (79.5)	70 (94.6)	
N category, *n*(%)^1^			0.001*
N0	68 (58.1)	31 (41.9)	
N1	38 (32.5)	20 (27.0)	
N2	11 (9.4)	23 (31.1)	
M category, *n*(%)^1^			< 0.001*
M0	113 (96.6)	60 (81.1)	
M1	4 (3.4)	14 (18.9)	
AJCC Stage, *n*(%)^1^			< 0.001*
I	21 (17.9)	1 (1.4)	
II	46 (39.3)	28 (37.8)	
III	46 (39.3)	31 (41.9)	
IV	4 (3.4)	14 (18.9)	
Differentiation, *n* (%)^1^			0.017*
Well	61 (52.1)	27 (36.5)	
Moderate	44 (37.6)	29 (39.2)	
Poorly	12 (10.3)	18 (24.3)	
Vessel invasion, *n* (%)^1^			0.042*
No	112 (95.7)	65 (87.8)	
Yes	5 (4.3)	9 (12.2)	

*P* values are based by ^1^Chi-square and ^2^Fisher's exact test.

* Significant associations between 2 categorical variables.

### Survival analysis and prognosis significance of BRG1 expression in colon cancer

**Figure 2:**
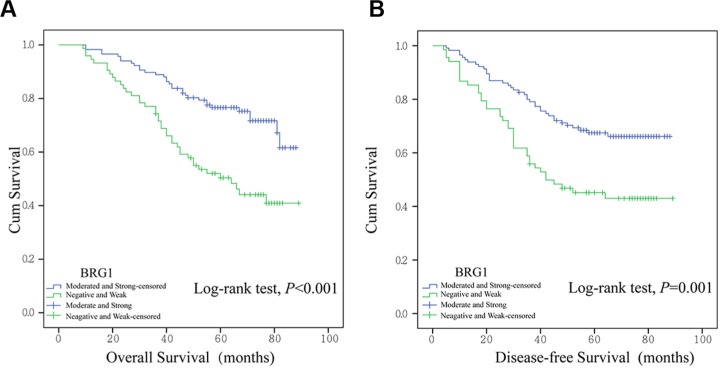
Kaplan-Meier survival analysis in 191 colon cancer patients Overall survival (**A**) and disease-free survival (**B**) were analyzed in patients with different BRG1 expression.

In order to assess the association between BRG1 expression and patient survival, we performed Kaplan-Meier curves with a log-rank test for overall survival (OS) and disease-free survival (DFS). Significant differences in OS and DFS between two expression levels were observed. Higher BRG1 expression leads to reduction of overall survival (Figure 2A, Overall Survival, *P* < 0.001) and earlier tumor occurrence after surgery (Figure 2B, Disease-free Survival, *P* = 0.001).

Furthermore, Cox proportional hazards model univariate and multivariate analysis were undertaken to exam the prediction value of BRG1 in patient prognosis after surgery. On univariate analysis, patients whose localized colon tumors were BRG1-moderate/strong had a significantly lower overall survival (OS) than those with BRG1-negative/weak tumors (Table 2, hazard ratio [HR]: 2.494, 95% confidence interval [CI]: 1.564–3.975, *P* < 0.001). Patients with higher BRG1 expression had more than two-fold occurrence burden than those with lower BRG1 expression (Table 3, HR: 2.106, CI: 1.342–3.306, *P* = 0.001). In addition, the AJCC stage, T classification, presence of nodal involvement, metastatic disease, vessel invasion, and level of tumor differentiation were associated with OS and DFS (Table 2, Table 3). Multivariate analysis was performed using the Cox proportional hazards model for all of the significant variables in the univariate analysis. However, T stage, lymph node metastasis, and distant metastasis were collinear with AJCC stage, and we excluded them from the final model. The results showed that the prognosis of patients after surgery was highly correlated with AJCC stage and differentiation. Although BRG1 failed in acting as an independent prognosis factor in multivariant analysis (barely detectable statistically significant difference, Table 2, P = 0.074; Table 3, P = 0.055), there is a tendency for BRG1 playing an independent predictor of OS and DFS. If the sample enlarged, some significant results might be generated.

**Table 2:** Cox proportional hazards model univariate and multivariate analyses of individual parameters for correlations with overall survival (OS) in 191 patients

Variable	Univariate		Multivariate	
	HR (95% CI)	*P* value	HR (95% CI)	*P* value
Age				
< 65y	–		NR	
≥ 65y	0.911 (0.569–1.460)	0.699		
Gender				
Male	–		NR	
Female	1.390 (0.855–2.259)	0.184		
Location				
Right	–		NR	
Transverse	0.842 (0.348–2.035)	0.703		
Left	0.870 (0.380–1.993)	0.742		
Sigmoid	1.091 (0.654–1.821)	0.738		
T stage				
T1 and T2	–		NR	
T3 and T4	5.261 (1.654–16.738)	0.005*		
N stage				
N0	–		NR	
N1	4.523 (2.290–8.559)	< 0.001*		
N2	15.137 (7.901–28.998)	< 0.001*		
M stage				
M0	–		NR	
M1	14.616 (8.046–26.549)	< 0.001*		
AJCC stage				
I and II	–		–	
III and IV	7.484 (4.078–13.736)	< 0.001*	5.416 (2.872–10.214)	< 0.001*
Differentiation				
Well	–		–	
Moderate	2.425 (1.348–4.361)	0.003*	1.816 (0.997–3.306)	0.051
Poorly	7.587 (4.084–14.092)	< 0.001*	4.039 (2.052–7.947)	< 0.001*
Vessel invasion				
No	–		–	
Yes	4.572 (2.482–8.421)	< 0.001*	1.424 (0.742–2.731)	0.288
BRG1 expression				
Negative and weak	–		–	
Moderate and strong	2.494 (1.564–3.975)	< 0.001*	1.576 (0.957–2.595)	0.074

Abbreviations: AJCC, American Joint Committee on Cancer;BRG1, Brahma-related Gene 1.

*HR* Hazard ratio, *CI* confidence interval. *NR* variable was not included in the resultant model.

*P* < 0.05 indicated that the 95%CI of HR was not including 1.

**Table 3:** Cox proportional hazards model univariate and multivariate analyses of individual parameters for correlations with disease-free survival (DFS) in 191 patients

Variable	Univariate		Multivariate	
	HR (95% CI)	*P* value	HR (95% CI)	*P* value
Age				
< 65y	–		NR	
≥ 65y	0.912 (0.576–1.445)	0.695		
Gender				
Male	–		NR	
Female	1.151 (0.726–1.823)	0.549		
Location				
Right	–		NR	
Transverse	0.855 (0.355–2.061)	0.728		
Left	0.826 (0.362–1.885)	0.649		
Sigmoid	1.228 (0.748–2.015)	0.417		
T stage				
T1 and T2	–		NR	
T3 and T4	4.195 (1.532–11.489)	0.005*		
N stage				
N0	–		NR	
N1	2.970 (1.688–5.226)	< 0.001*		
N2	10.530 (5.875–18.875)	< 0.001*		
M stage				
M0	–		NR	
M1	9.772 (4.818–19.823)	< 0.001*		
AJCC stage				
I and II	–		–	
III and IV	4.559 (2.746–7.570)	< 0.001*	3.545 (2.079–6.043)	< 0.001*
Differentiation				
Well	–		–	
Moderate	2.242 (1.324–3.797)	0.003*	1.813 (1.061–3.097)	0.029*
Poorly	4.756 (2.552–8.861)	< 0.001*	2.947 (1.502–5.781)	0.002*
Vessel invasion				
No	–		–	
Yes	3.994 (2.088–7.639)	< 0.001*	1.481 (0.730–3.007)	0.277
BRG1 expression				
Negative and weak	–		–	
Moderate and strong	2.106 (1.342–3.306)	0.001*	1.588 (0.990–2.548)	0.055

Abbreviations: AJCC, American Joint Committee on Cancer; BRG1, Brahma-related Gene 1.

*HR* Hazard ratio, *CI* confidence interval. *NR* variable was not included in the resultant model.

*P* < 0.05 indicated that the 95%CI of HR was not including 1.

### Knocking down BRG1 suppresses colon cancer cell proliferation and invasion

**Figure 3:**
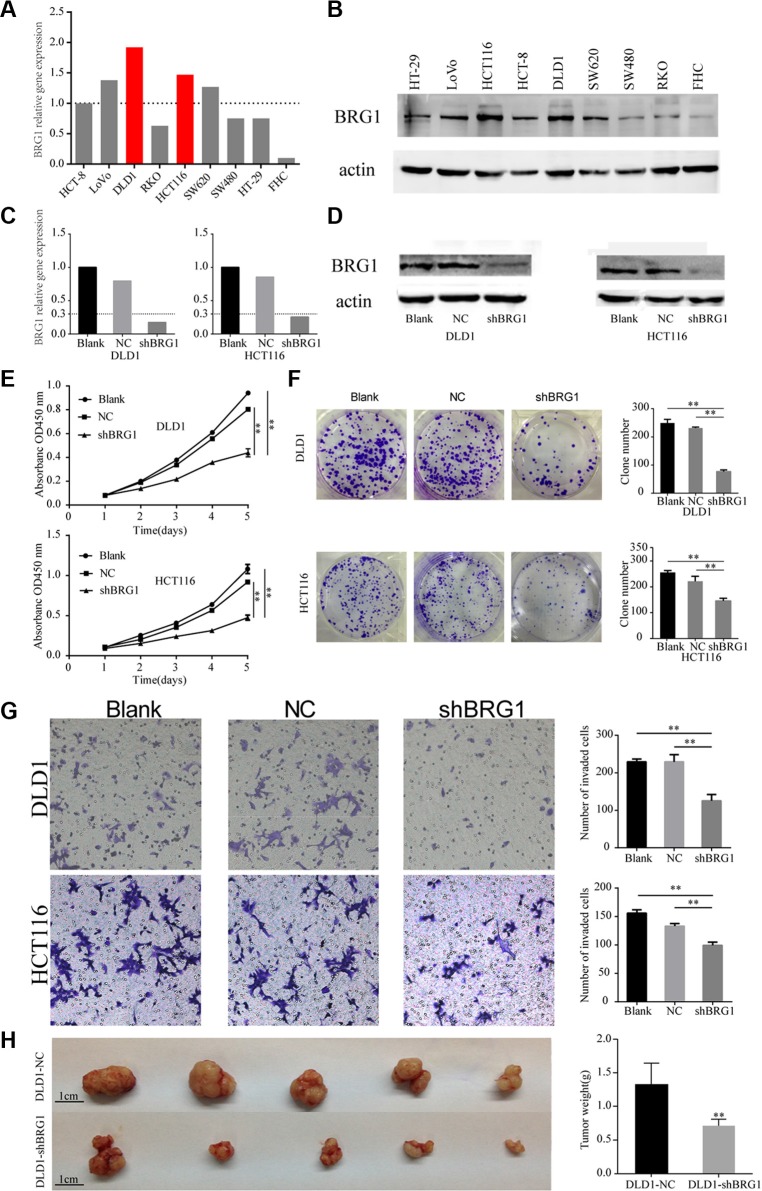
Knocking down BRG1 expression in colon cancer cells inhibits cell proliferation and invasion (**A**) Real-time PCR analysis of different BRG1 expression in eight colon cancer cell lines and one normal epithelium cell line. BRG1 expression in HCT8 was set as reference. (**B**) Western Blot analysis of different BRG1 expression in eight colon cancer cell lines and one normal epithelium cell line. (**C**) Real-time PCR analysis of BRG1 mRNA in cells after RNA interference. (**D**) Western Blot analysis of BRG1 protein in cells after RNA interference. (**E**) CCK-8 cell proliferation assays revealed the impact of BRG1 on cell proliferation in DLD1 and HCT116 cells (**P < 0.01). (**F**) Clone formation assays revealed the impact of BRG1 on clone formation ability in DLD1 and HCT116 cells (**P < 0.01). (**G**) Transwell invasion assays revealed the impact of BRG1 on invasion ability in DLD1 and HCT116 cells (200×, **P < 0.01). (**H**) Xenograft model revealed the impact of BRG1 on proliferation *in vivo* in DLD1 cells (bar = 1 cm, **P < 0.01).

Since higher BRG1 expression correlated with advanced tumor stage and poor prognosis, we decided to explore the effect of BRG1 on tumor progression in cell lines. BRG1 expression is abundant in HCT116 and DLD1 cells at both mRNA and protein levels (Figure 3A and 3B), and the two cell lines were of high degree in malignancy. Thus, lentivirus-mediated RNA interference was performed on the two cell lines to suppress BRG1 expression. After RNA interference, Real-time PCR and Western Blot were performed to evaluate the interference efficiency. The results showed that BRG1-specific shRNA successfully silenced the expression of BRG1 48 h after transfection in HCT116 and DLD-1 cells (Figure 3C and 3D). Knocking down BRG1 expression significantly inhibited colon cancer cell proliferation (Figure 3E, **P < 0.01), cancer cell clonogenicity (Figure 3F, **P < 0.01) and invasive ability (Figure 3G, **P < 0.01). These results suggest that BRG1 plays a key role in promoting colon cancer cell proliferation and invasion.

### BRG1 promotes tumor growth *in vivo*

To evaluate the effect of BRG1 on tumorigenesis *in vivo*, shRNA-BRG1 or negative control-transfected DLD1 cells were subcutaneously implanted in mice. After 21 days feeding, the xenograft were excised. As shown in Figure 3H, the volume of tumor formed from DLD1-shBRG1 was smaller than the tumor formed from control cells. Moreover, the average weight of shRNA-BRG1 tumors was markedly lighter as compared to controls (1.32 ± 0.33 g vs. 0.69 ± 0.11 g, *P* = 0.001, Figure 3H). Study results revealed that BRG1 positively regulate cell proliferation in colon cancer *in vivo* models, which coincided with the results observed in the *in vitro* models.

### Association between BRG1 expression and WNT3A expression pattern

**Figure 4:**
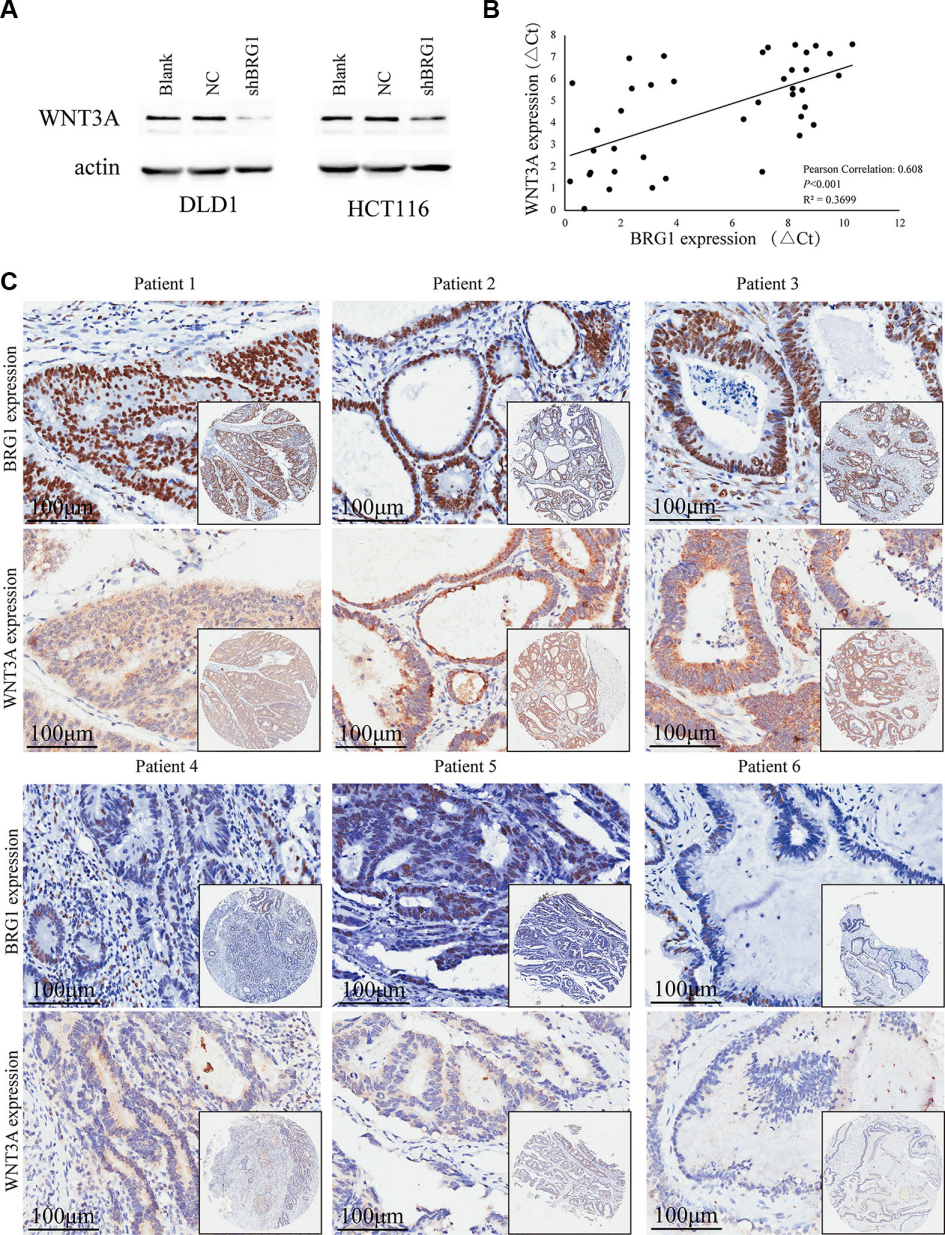
BRG1 expression correlates with WNT3A expression positively (**A**) Western Blot analysis of WNT3A expression in DLD1 and HCT116 cells after BRG1 RNA interference. (**B**) Positive correlation between BRG1 mRNA levels and WNT3A mRNA levels in 40 colon cancer tissues (Pearson Correlation: 0.608, *P* < 0.001, R^2^ = 0.3699). (**C**) Immunohistochemial staining revealed positive correlation between BRG1 protein levels and WNT3A protein levels in tissue microarrays.

In order to elucidate how BRG1 regulates colon cancer progression, we made the decision to investigate the potential targets directly regulated by BRG1. In previous work, we identified WNT3A as a potential downstream gene of BRG1 by combining microarrays and bioinformatics analysis. Western blot analysis revealed that knocking down BRG1 expression inhibits WNT3A expression in both DLD1 and HCT116 cells (Figure 4A).

Real-time PCR and immunohistochemistry were conducted to assess the expression of BRG1 and WNT3A in 40 fresh colon cancer tissues and consecutive tissue microarrays, respectively. The expression of WNT3A mRNA has notable positive correlation with BRG1 in colon cancer tissues (Figure 4B, Pearson Correlation:0.608, P < 0.001, R2 = 0.3699). Immunohistochemical staining also showed similar results that patients whose localized colon tumors were BRG1-strong had a significantly higher WNT3A expression than those with BRG1 negative/weak tumors. Six representative IHC results of consecutive sections were shown in Figure 4C. These results accord with our previous work, and WNT3A is highly likely regulated by BRG1 in colon cancer cells.

### Restoration of WNT3A expression rescues the inhibition of proliferation and invasion ability

**Figure 5:**
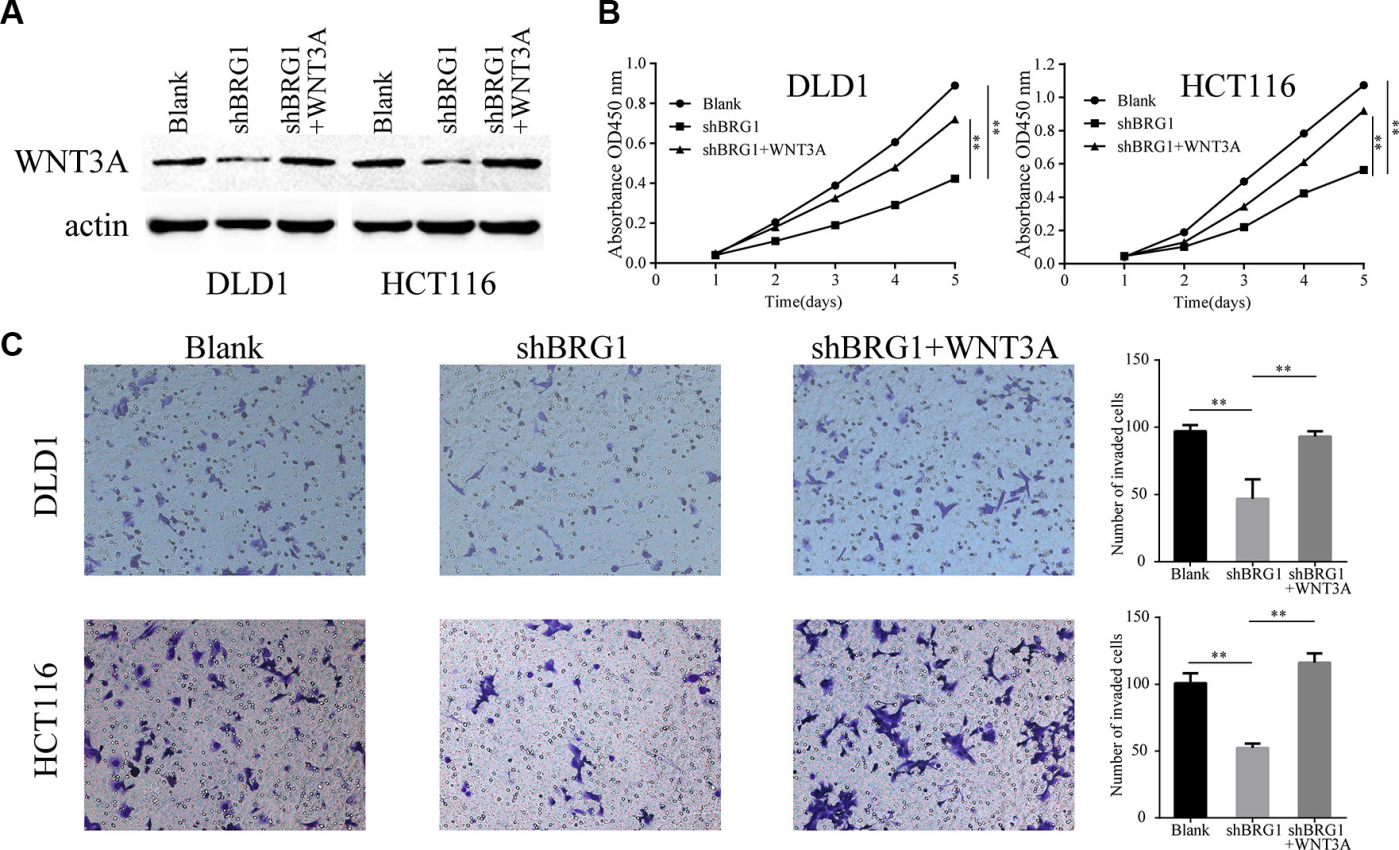
BRG1 regulates colon cancer cell proliferation and invasion via positive regulation of WNT3A (**A**) Western Blot analysis WNT3A protein in DLD1 and HCT116 cells after restoration of WNT3A expression. (**B**) CCK-8 proliferation assays revealed the impact of WNT3A on cell proliferation in BRG1 knocked down cells (**P < 0.01). (**C**) Transwell invasion assays revealed the impact of WNT3A on invasion ability in BRG1 knocked down cells (200×, **P < 0.01).

WNT3A has been implicated in oncogenesis in several tumors [22] and is associated with proliferation and invasion in colon cancer cells [20]. In order to assess whether WNT3A participates in the promotion of BRG1-induced malignant phenotype, BRG1 knocked down cells were transfected by the lentivirus vector expressing WNT3A (Figure 5A). CCK-8 proliferation assays were performed and the results showed that restoration of WNT3A expression rescues the inhibition of proliferation ability (Figure 5B, **P < 0.01). In addition, restoration of WNT3A expression promotes invasion in colon cancer cells (Figure 5C, **P < 0.01). These results demonstrate that BRG1 could promote colon cancer progression via positive regulation of WNT3A.

## DISCUSSION

The mammalian SWI/SNF complexes constitute a family of chromatin remodeling proteins that regulate gene expression by disrupting histone-DNA contacts in an ATP-dependent manner [23]. The BRG1, which is a central ATPase subunit of SWI/SNF complexes, is crucial for the involvement of proliferation and differentiation [24, 25]. Previous studies have reported vital and various roles of BRG1 in several cancers, including prostate cancer, breast cancer, lung cancer, melanomas, and others [10, 16, 26, 27]. The biological significance of BRG1 differs during the pathogenesis of human cancer according to the cell and/or tissue type.

Watanabe et al. reported overexpression of BRG1 in colorectal mucosa, adenoma, and carcinoma with an upward trend by analyzing BRG1 and BRM expression in 31 patients [28]. But the relationship between BRG1 and clinical features, the prognosis value of BRG1 remained unclear. In the present study, we recorded the clinical pathological significance of BRG1 expression in human colon cancer. First, we confirmed the presence of increased expression of BRG1 at both mRNA and protein levels in two independent tissue groups, which is in accordance with a prior study [28]. Second, comprehensive analysis of clinical data and BRG1 expression indicated that the increased expression of BRG1 was associated with AJCC stage, depth of tumor invasion, distant metastasis, and histologic differentiation in colon cancer. The results indicated a high malignant degree in colon cancer with elevated BRG1 expression. Although BRG1 failed to act as an independent prognosis predictor on multivariate analysis, the Kaplan–Meier curve for DFS and OS revealed a poorer prognosis in patients with higher BRG1 expression. Hence, in collaboration with other biomarkers, BRG1 expression levels could be taken into consideration when making treatment decisions. Patients with elevated BRG1 expression requires more powerful adjunctive treatments and frequent postoperative follow-up, which serves as a profound guide in clinical treatment.

Previous studies focused on the effect of BRG1 on cell biological behavior, and the results varied due to the differences between types of tissues/cells. In lung tissue, BRG1 promotes cell differentiation and prevents tumorigenesis by antagonizing Myc activity [29]. In the immune system, BRG1 regulates B cell activation through pariticipating in widespread gene expression and cell proliferation [30]. In cancer, experiments *in vitro* have revealed that BRG1 accelerates cancer cell proliferation in glioma and in breast cancer [16, 31]. Nevertheless, BRG1 inhibits cancer aggressiveness, acting as tumor suppressor in NSCLC cells [10]. The different originations and genetic backgrounds may generate to the discrepancy. On the other hand, previous research reported the diverse roles of BRG1 in several cancer types [32]. BRG1 may participate in different biological processes according to different cancer types. In our research, experiments in two colon cell lines and assays *in vivo* came to the same conclusion that elevated BRG1 expression promotes colon cancer proliferation and invasion. These findings correspond with the poor prognosis in patients with high BRG1 expression. Considering that it promotes the aggressiveness in colon cancer, BRG1 may be a potential therapy target for drug development. Antagonizing BRG1 may inhibit colon cancer progression and prolong patient survival.

Previous research has shown that BRG1 participates in diverse processes through various ways. Several studies reported BRG1 promotes cancer cell proliferation by cooperation with CBP or regulating cyclin D1 and cyclin E expression [16, 31, 33]. Saladi et al. reported that BRG1 modulates MMP2 expression contributing melanoma invasiveness [27], while Sanchez-Tillo et al. showed that BRG1 promotes tumor invasion by regulating E-cadherin expression and epithelial-to-mesenchymal transition [34]. In previous study, we identified WNT3A as a potential downstream gene of BRG1 by gene expression microarray and bioinformatics analysis. In this study, we demonstrated that BRG1 activates WNT3A expression in colon cancer cells. Growing evidence demonstrates that BRG1 regulates a widespread cell processes through remodeling nucleosomes and modulating transcription. Increased WNT3A expression has been reported in association with clinical stage, malignant behavior in several cancers [20, 35, 36]. Whether BRG1 promotes colon cancer progression through positive regulation of WNT3A is still unknown. In this study, we revealed a positive correlation between BRG1 and WNT3A expression in colon cancer tissues and cell lines. After restoration of WNT3A expression in BRG1 knocked down cells, the inhibition of proliferation and invasion ability was rescued.

However, a previous study suggested that BRG1 regulates the cell cycle and cell proliferation by activating the PI3K-Akt signaling pathway in colon cancer [28]. The different results can be explained by the genetic background discrepancy of two cell lines and different bioinformatic analysis method. On the other hand, it’s of great possibility that BRG1 plays diverse roles in colon cancer and regulates biological behavior through various approaches. Diverse roles of BRG1 were obtained through a global approach and gradually increasing evidence deepened our understanding of BRG1 [32]. Recent studies have displayed that BRG1 is involved in chromatin structure variation, dynamic position of nucleosomes, and accessibility of gene promoters [37]. It has been proven that BRG1 binds at distal regulatory sequences and acts as a dual function in tissue-specific gene regulation during early mammalian development [38]. In the future, chromatin structure variations affected by BRG1 in colon cancer should be assessed, and the precise binding sequences in target genes of BRG1 will be addressed.

In conclusion, we identified BRG1 as an important stage-associated protein in colon cancer. Increased BRG1 expression may have an important role in promoting tumorigenesis. Moreover, it is closely correlated with aggressive malignant behavior and its presence predicts poor survival for colon cancer patients. In addition, BRG1 contributes to colon cancer proliferation and invasion through positive regulation of WNT3A expression. Our research provides a new understanding of colon cancer progression and may have general implications in the biology of cancer. In the future, larger clinical sample validation and more research aimed at the mechanism of BRG1 are required to fully elucidate its role in colon cancer progression.

## MATERIALS AND METHODS

### Human tissue specimens and tissue microarray information

The custom colon cancer tissue TMA (191 cases, collected from Shanghai General Hospital) and a commercial TMA (75 cases, purchased from Outdo Biotech, Shanghai, PR China) were used to detect BRG1 protein expression through immunohistochemical analysis. 191 specimens from our hospital were collected and archived by protocols that were approved by the institutional review boards of the Shanghai General Hospital. Informed consents were obtained from all the patients. 173 patients with colon cancer at stage I, II and III underwent the radical resection of colon cancer and 18 patients with colon cancer and synchronous metastatic liver tumor (IV stage) received simultaneous resection of colon cancer and liver metastasis. This group includes 78 male and 113 female with a mean age of 66 (range, 22–95) years. The diagnoses were conducted by at least two pathologists who were blind to the patients’ information. The follow-up were performed according to the National Comprehensive Cancer Network Practice guidelines and the end date of the follow-up was June 29, 2008.

### Immunohistochemistry

Immunohistochemical staining was carried out using a rabbit anti-BRG1 polyclonal antibody (sc-10768, Santa Cruz, USA, diluted 1:250) and a rabbit anti-WNT3A polyclonal antibody (ab19925, Abcam, Cambridge, United Kingdom, diluted 1:100). Positive staining was scored by two independent investigators who had no knowledge of the patient outcomes. When discrepancy in an assessment was encountered, the slides were re-examined by both pathologists under a multi-head microscope to obtain agreements. The staining intensity was scored as being 0 (negative), 1 (weak), 2 (moderate), or 3 (strong). The extent of staining was scored according to the percentage of positively stained cells: 0 (0–10%), 1 (11–25%), 2 (26–75%), and 3 (75–100%). The sum of the intensity and extent of the scores were defined as the final staining score. The specimens were divided into 2 groups according to their overall scores, which were as follows: 0–3 = negative and weak, 4–6 = moderate and strong positive.

### Cell lines and culture conditions

The human colon cancer cell lines were obtained from the Cell Resource Center of the Shanghai Institutes for Biological Sciences Type Culture Collection of the Chinese Academy of Sciences (Shanghai, PR China). And the normal human colon epithelial cell lines, FHC, was purchased from ATCC. All of the cells were maintained at 37°C in 5% CO2 in Dulbecco’s modified Eagle’s medium and was supplemented with 10% FBS (Gibco; Carlsbad, CA, USA).

### Western blot

Total proteins were extracted from cultured cells and tissues using RIPA lysis buffer (Beyotime Biotechnology, Jiangsu, China). Protein concentrations were determined using BCA protein assay kit (Beyotime Biotechnology, Jiangsu, China). Equal amounts of protein were separated by electrophoresis on SDS polyacrylamide gel and transferred onto polyvinylidene difluoride membranes. The membranes were blocked with 5% non-fat milk solution for 1 h, and then incubated with a primary antibody overnight at 4°C and a secondary antibody at room temperature, successively. The bands were detected by ECL chemiluminescence (Millipore, USA) according to the manufacturer’s instructions. Expression of GAPDH or actin was applied as internal control to confirm equal loading of whole protein.

Antibodies used in the Western blot assay were as follows: anti-BRG1 polyclonal antibody (sc-10768, Santa Cruz, USA, diluted 1:250), anti-WNT3A polyclonal antibody (ab19925, Abcam, Cambridge, United Kingdom, diluted 1:100), anti-GAPDH antibody (sc-137179, Santa Cruz; USA, diluted 1:2000), anti-actin antibody (60008-1-Ig, Proteintech; USA, diluted 1:2000).

### Real-time polymerase chain reaction

Total RNA was extracted with an RNeasy kit (Qiagen, Germany) and single-stranded cDNAs were synthesized with a High Capacity cDNA Reverse Transcription kit (Applied Biosystems). qPCRs were carried out with SYBR Green PCR Master Mix (Applied Biosystems) and Mastercycler^®^ ep realplex (Eppendorf, Germany). The housekeeping gene GAPDH was applied as an internal control. The primer sequences were as follows: BRG1 (forward, 5′-TGAGAATGCCAAGCAAGATG-3′ and reverse, 5′-AGGATGCCGTTCAGGTTGT-3′), WNT3A (forward, 5′-TCCCACGTACTCCAACTTCCA-3′ and reverse, 5′-AGCACCAGAAACACGTGCACT-3′).

### Lentiviral-mediated RNA interference and overexpression

A lentiviral vector encoding a short hairpin RNA (shRNA) targeted against BRG1 and a negative control lentiviral vector were constructed and transfected into 293T cells. The lentiviruses were collected to transfect DLD1 and HCT116 cells. The WNT3A gene (CDS region sequence) was synthesized according to the human WNT3A gene’s mRNA sequence and cloned into the lentiviral vector. Recombinant lentiviruses were produced after the 293T packing cells were co-transfected with lentiviral vector and lentiviral packaging plasmids. Cell lines for stable expression of WNT3A were established by incubating BRG1 knockdown cells with the infectious lentivirus medium of lenti-WNT3A. Western blot was performed to analyze the protein expression of BRG1 or WNT3A in cells after 48 h. The lentiviral plasmids used for sh-BRG1 and WNT3A transfer were LV12 and LV6 (Genepharma, Shanghai), respectively. And the negative control lentiviral vector targeting none gene was purchased from Genepharma, Shanghai.

### Cell proliferation assay and clone formation assay

Exponentially growing cells were re-suspended and seeded in 96-well plates at a density of 500 cells per well. The cells were incubated for 7 days. Ten microliter of CCK-8 solution was added to the detection well. Cell viability was determined by measuring the absorbance at 450 nm after cultured for 2 h. Clone formation was determined by preparing single cell suspension solutions and seeding in 6-well plate with 500 cells/well. After cells incubated for 14 days, the colonies were stained with crystal violet for 20 min. Stained clones were counted and the plates were photographed. All experiments were independently repeated triplicate times.

### Transwell invasion assay

For the invasion assay, the BD Matrigel Invasion Chamber was used according to the manufacturer’s protocol. Cells (5 × 10^4^) were suspended in serum-free DMEM and seeded on matrix membranes. DMEM supplemented with 20% FBS was added to the lower chamber. After 24 h incubation, the cells that had penetrated the Transwell chamber were fixed with 4% paraformaldehyde and stained with crystal violet for 20 min. The number of cells was counted using an inverted microscope at 20 high-power. All photographs were taken under high magnification with a microscope (Nikon, Tokyo, Japan).

### Xenograft model

Animal studies were carried out in accordance with the guidelines that were established by the Shanghai Resource Center of Laboratory Animals of the Chinese Academy of Science. Five 4 to 6-week-old (18–20 g) BALB/c nu/nu mice per group were subcutaneously injected in the right axilla with 4 × 10^6^ cells that were suspended in 0.2 mL of PBS. The mice were sacrificed on Day 21 after the tumors had been implanted, and the tumor weights and volumes were quantified. A sliding caliper was used to measure the sizes of the tumors, and the volumes of the tumors were calculated using the following formula: width2 × length × 0.5.

### Statistical analysis

The Chi-square/Fisher’s exact test for proportionality was used to analyze the relationship between the expression of BRG1 and the clinicopathological variables. The survival rates were calculated by the Kaplan-Meier method, and the differences between the survival curves were examined by the log-rank test. Univariate Cox proportional hazards regressions were applied to estimate the individual hazard ratio (HR) for the disease-free survival (DFS) and overall survival (OS). The significant variables in the univariate analyses (*P* < 0.05) were then put into the multivariate analysis. The HR with 95% confidence interval was measured to estimate the hazard risk of individual factors. Continuous variables between the 2 groups were compared by an independent two sample *t*-test. Data are displayed as mean and standard deviation. A *P*-value of less than 0.05 was considered to be statistically significant. All statistical analyses were carried out using the SPSS 19.0 statistical software package (SPSS Inc, Chicago, IL).

## SUPPLEMENTARY MATERIALS TABLES


