# Supplementary material for: The chromatin-remodeling enzyme BRG1 promotes colon cancer progression via positive regulation of WNT3A
Source: Oncotarget. 2016 Nov 12;7(52):86051–63. doi: 10.18632/oncotarget.13326 (PMC5349896; doi:10.18632/oncotarget.13326)
Supplement: Supplementary file 1 [file oncotarget-07-86051-s001.pdf]

The chromatin-remodeling enzyme BRG1 promotes colon cancer progression via positive regulation of WNT3A

Supplementary Materials

Supplementary Table S1: Associations of BRG1 expression with clinicopathological features in colon cancer (n = 75)

Variables	BRG1 expression		*P* value*
	Negative and weak (n = 28)	Moderate and Strong (n = 47)	
Age, n (%)[1]			0.833
< 65 years	15 (53.6)	24 (51.1)	
≥ 65 years	13 (46.4)	23 (48.9)	
Gender, n (%)[1]			0.069
Male	18 (64.3)	20 (42.6)	
Female	10 (35.7)	27 (57.4)	
Location, n (%)[2]			0.427
Right	16 (57.1)	23 (48.9)	
Transverse	4 (14.3)	4 (8.5)	
Left	8 (28.6)	20 (42.6)	
T category, n (%)[2]			0.047*
T1	1 (3.6)	2 (4.3)	
T2	7 (25.0)	2 (4.3)	
T3	17 (60.7)	32 (68.1)	
T4	3 (10.7)	11 (23.4)	
Lymph node metastasis, n (%)[1]			0.069
Negative	18 (64.3)	20 (42.6)	
Positive	10 (35.7)	27 (57.4)	
M category, n (%)[2]			0.470
M0	26 (92.9)	40 (85.1)	
M1	2 (7.1)	7 (14.9)	
AJCC Stage, n (%)[2]			0.020*
I	9 (32.1)	3 (6.4)	
II	9 (32.1)	14 (29.8)	
III	8 (28.6)	23 (48.9)	
IV	2 (7.1)	7 (14.9)	
Differentiation, n (%)[2]			0.657
Well	4 (14.3)	6 (12.8)	
Moderate	19 (67.9)	28 (59.6)	
Poorly	5 (17.9)	13 (27.7)	

P values are based by 1Chi-square and 2 Fisher's exact test.
* Significant associations between 2 categorical variables.
